# RIPK1-Associated Inborn Errors of Innate Immunity

**DOI:** 10.3389/fimmu.2021.676946

**Published:** 2021-06-07

**Authors:** Jiahui Zhang, Taijie Jin, Ivona Aksentijevich, Qing Zhou

**Affiliations:** ^1^ The Key Laboratory of Biosystems Homeostasis & Protection of Ministry of Education, Life Sciences Institute, Zhejiang University, Hangzhou, China; ^2^ Liangzhu Laboratory, Zhejiang University Medical Center, Hangzhou, China; ^3^ Inflammatory Disease Section, National Human Genome Research Institute, National Institutes of Health, Bethesda, MD, United States

**Keywords:** RIPK1, programmed cell death, immunodeficiency, autoinflammatory disease, CRIA, biological therapies

## Abstract

RIPK1 (receptor-interacting serine/threonine-protein kinase 1) is a key molecule for mediating apoptosis, necroptosis, and inflammatory pathways downstream of death receptors (DRs) and pattern recognition receptors (PRRs). RIPK1 functions are regulated by multiple post-translational modifications (PTMs), including ubiquitination, phosphorylation, and the caspase-8-mediated cleavage. Dysregulation of these modifications leads to an immune deficiency or a hyperinflammatory disease in humans. Over the last decades, numerous studies on the RIPK1 function in model organisms have provided insights into the molecular mechanisms of RIPK1 role in the maintenance of immune homeostasis. However, the physiological role of RIPK1 in the regulation of cell survival and cell death signaling in humans remained elusive. Recently, RIPK1 loss-of-function (LoF) mutations and cleavage-deficient mutations have been identified in humans. This review discusses the molecular pathogenesis of RIPK1-deficiency and cleavage-resistant RIPK1 induced autoinflammatory (CRIA) disorders and summarizes the clinical manifestations of respective diseases to help with the identification of new patients.

## Introduction

RIPK1 is a key modulator of inflammatory signaling and cell death pathways and its functions ultimately determine the cell fate upon different cellular stimulations. A growing body of evidence suggests that many human diseases, including systemic inflammation (1–5), cardiovascular disease (6), neurodegenerative disease (7), and cancer (8) are related to dysregulations in RIPK1-mediated cell death pathways. In recent years, multiple studies in model organisms have provided better understandings of the intricate biological functions of RIPK1. However, the role of RIPK1 in the regulation of immune responses has not been fully understood until very recently with the identification of RIPK1-deficiency Immunodeficiency 57 With Autoinflammation; OMIM: # 618108 (9–12) and the autoinflammatory disease named CRIA (Cleavage-resistant RIPK1-Induced Autoinflammatory) (13, 14). Both disorders are characterized by a profound dysregulation of cytokine expression and cell death pathways, and severe immune dysfunction.

## RIPK1 as a Regulator of Cell Death Pathways

Apoptosis and necroptosis are two distinct forms of programmed cell death. Both are critical for the regulation of embryonic development, immune response, and many other biological processes (15, 16). Apoptosis is executed by caspase-mediated events, while necroptosis is mediated by RIPK3 (receptor-interacting serine/threonine-protein kinase 3), MLKL (mixed lineage kinase domain-like protein) and upstream effectors such as RIPK1. The upstream regulator, RIPK1, is a key signaling node for determination of cell fate in response to TNF stimulation.

**Figure 1:**
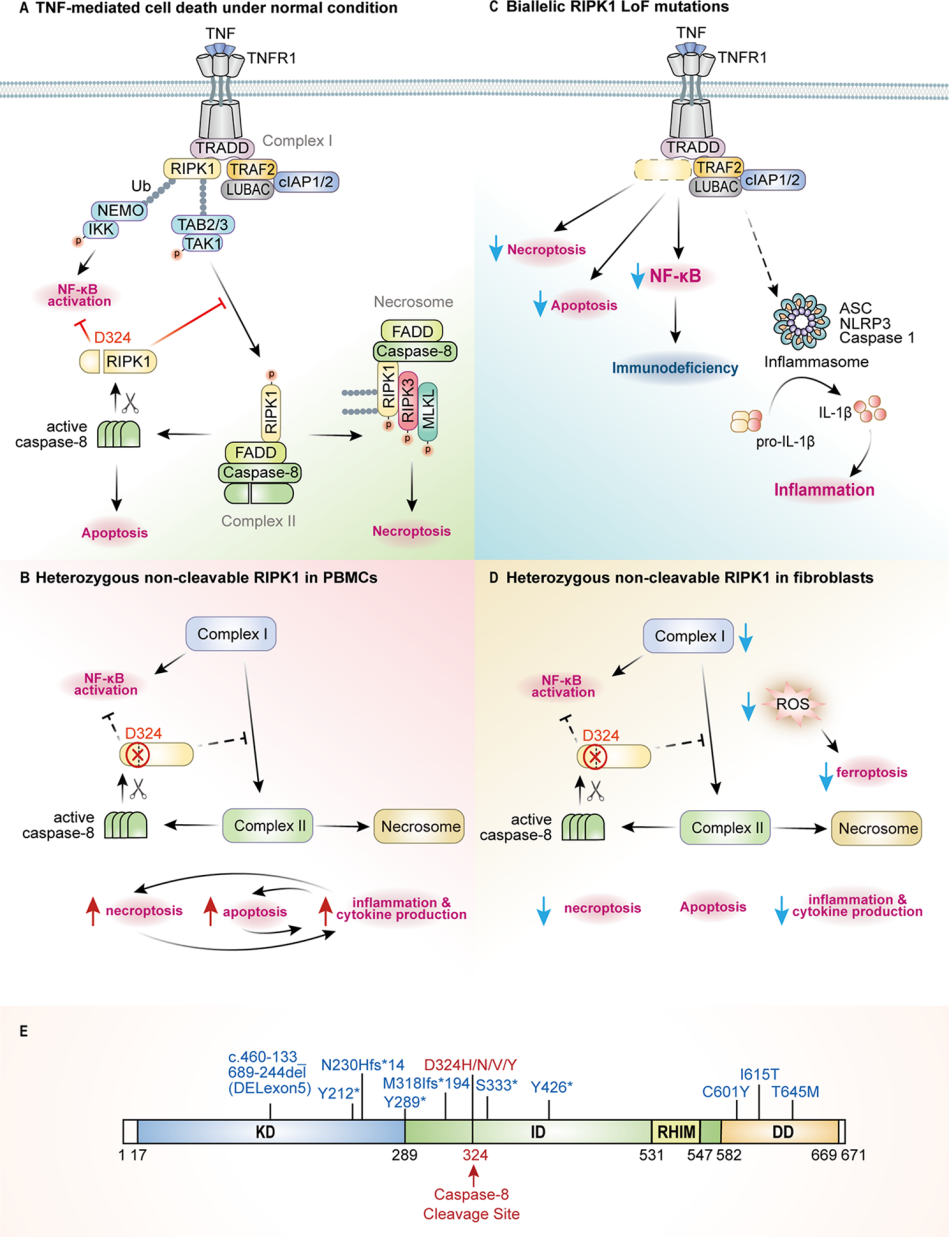
The pathogenic mechanisms of RIPK1-deficiency and CRIA patients. **(A)** RIPK1 is the key switch protein to mediate signal transduction downstream of TNFR1. TNF mediated TNFR1 trimerization results in recruitment of TRADD and RIPK1 through death-domain interactions to form complex I where post translational modifications (PTMs) by E3 ligases such as TRAFs, cIAPs and LUBAC or kinase complexes NEMO/IKK, TAK1/TAB2/3 determine the cell fate either to activate NF-κB signaling or to execute programmed cell death pathways. RIPK1 activates itself through *trans*-autophosphorylation and then interacts with FADD/caspase-8 to form the complex II to undergo RIPK1-dependent apoptosis (RDA), or it binds RIPK3/MLKL to form the necrosome and to initiate necroptosis. Active caspase-8 can cleave RIPK1 at Asp324 to inhibit complex II formation, and the RIPK1 inactivation will shift the balance towards necroptotic signaling. **(B)** In PBMCs of CRIA patients, due to the pathogenic mutations at Asp324, RIPK1 activation and all downstream signaling pathways are amplified to promote inflammation and cytokine production. **(C)** In case of the RIPK1 deficiency caused by biallelic LoF mutations, complex I-mediated signaling and downstream events will be blocked, leading to decreased NF-κB activation and impaired cell death. However, deficiency of RIPK1 may promote noncanonical TLR4-TRIF-RIPK1-FADD-Caspase-8 signaling to activate inflammasome formation and necroptosis. **(D)** Non-cleavable RIPK1 in CRIA patients fibroblasts causes reduced inflammatory signaling and necroptosis, but almost unchanged levels of apoptosis under TNFR1 signaling pathway. Besides, the decreased production of ROS in fibroblasts through yet unknown mechanisms leads to reduced ferroptosis. **(E)** The position of *RIPK1* (NM0038.4.6) LoF mutations (blue, homozygous) and cleavage-deficient mutations (red, heterozygous) are indicated across the protein domains.

RIPK1 is a 75.9 kDa protein with an amino-terminal kinase domain (KD), a carboxy-terminal death domain (DD), and an intermediate domain (ID) in-between which contains a Rip homotypic interaction motif (RHIM). RIPK1 plays a crucial role in mediating downstream signals triggered by DRs and PRRs, and that function is highly dependent on its PTMs, including ubiquitination, phosphorylation or cleavage. For example, the ubiquitination status of RIPK1 can trigger a switch from NF-κB-mediated pro-survival inflammatory signaling to cell death pathways *via* activation of caspase-8-dependent apoptosis or RIPK3/MLKL-dependent necroptosis (17–19). Binding of tumor necrosis factor (TNF) to TNF receptor 1 (TNFR1) on cell surface initiates receptor trimerization and recruitment of TRADD (TNFR1-associated death domain) and RIPK1 to form the signaling complex I. This complex then recruits a series of E3 ligases including TRAF2/5 (TNF receptor associated factor 2 and 5), cIAP1/2 (cellular inhibitor of apoptosis protein 1 and 2), and LUBAC (linear ubiquitin chain assembly complex) to ubiquitinate various components in the complex (20). This process provides a docking platform to recruit kinases including TAK1 (transforming growth factor β-activated kinase 1), IKKα/β/γ/ϵ (Inhibitor of κB kinase α/β/γ/ϵ) and TBK1 (TANK-binding kinase 1) to activate NF-κB and MAPK signaling pathways, which results in expression of proinflammatory cytokines such as IL-6 and IL-8, as well as promoting proliferation and cell survival (18–20) (**Figure 1A**). A set of kinases such as TAK1 (21), MK2 (22–24), IKKα/β (25), and TBK1 (26, 27) can phosphorylate RIPK1 at different sites to inhibit its kinase activity. The RIPK1 function is also negatively regulated by deubiquitinases (DUBs), including A20 (28) and CYLD (Cylindromatosis) (29, 30).

Under pathological conditions when NF-κB is inhibited or RIPK1 is no longer ubiquitinated or phosphorylated (18–20), RIPK1 interacts with FADD (FAS-associated death domain) and caspase-8 to form complex II, which then triggers downstream RIPK1-dependent apoptosis (RDA). In case where caspase-8 activation is blocked, RIPK1 auto-phosphorylates at Ser166 (31) and co-aggregates with RIPK3, through the RHIM-RHIM interaction, and MLKL to form the complex termed necrosome (32–35). Subsequently, MLKL gets phosphorylated by RIPK3 and causes necroptosis by disrupting the plasma membrane (36, 37). In complex II, caspase-8 serves as a regulatory switch of RIPK1 activity through cleavage at RIPK1 residue Asp324 (**Figure 1A**). Pathogenic genetic variants that block the cleavage of RIPK1 by caspase-8 lead to RIPK1 over-activation, and the promotion of RDA and necroptosis (13, 14, 38, 39) (**Figure 1B**). RIPK1 deficiency due to pathogenic LoF variants leads to reduced NF-κB activation and dysregulated cell death under specific conditions, e.g., TNF or poly(I:C) stimulation, or a combination of TNF plus BV6 (IAP inhibitor) induced apoptosis and TNF, BV6 plus zVAD-fmk (pan-caspase inhibitor) induced necroptosis in patients’ fibroblasts, as well as in *RIPK1* ^-/-^ HT-29 cell lines (9, 10) (**Figure 1C**).

The complicated mechanisms of RIPK1 in mediating inflammation and cell death have been described in numerous studies using mouse models. Constitutive *Ripk1^-/-^* mice die postnatally due to dysregulated cell death and systemic inflammation (40), which can be rescued by knockout of *Ripk3* and either *Fadd* or *Casp8* (1, 3–5). Because of the indispensable role of RIPK1 in embryonic development, conditional knockout mice were constructed to study cell-specific functions of this protein. *Ripk1* deletion in mouse intestinal epithelial cells (IECs) causes IEC apoptosis and loss of goblet and Paneth cells, manifesting with severe intestinal pathology and premature death (41, 42). Similarly, *Ripk1* deletion in epidermis cell induces keratinocyte apoptosis and necroptosis, resulting in severe skin inflammation (42). In contrast to the phenotype of constitutive and conditional *Ripk1* ^–/–^ mice, mice engineered with RIPK1 knock-in (KI) kinase-dead mutations, including Asp138Asn (43), Lys45Ala (5, 44), Gly27_Phe28del (45) and the Lys584Arg (46) mutation affecting the dimerization of death domain, are viable and normal, which indicates the dispensable role of RIPK1 kinase activity in regulating development.

Caspase-8 cleavage activity is essential for inhibition of the RIPK1-dependent cell death during embryogenesis. *Casp8* knockout in mice leads to embryonic lethality due to defects in the vascular, cardiac, and hematopoietic systems (47). This phenotype is linked to increased necroptosis and can be rescued by the deletion of either *Ripk3* or *Mlkl* (48–50). Similarly, mice with enzymatically inactive caspase-8 (Cys362Ser or Cys362Ala) also died at embryonic stage owing to increased endothelial cell necroptosis that can be prevented by knockout of *Mlkl*. However, lethality of *Casp8^C362S/C362S^Mlkl^–/–^* mice was noted at perinatal stage (38, 51). Furthermore, conditional expression of inactive caspase-8 in IECs caused ASC [apoptosis-associated speck-like protein containing a caspase-activation and recruitment domain (CARD)] assembly, while loss of either caspase-1 or ASC helped the normal development of *Casp8^C362S/C362S^Mlkl^–/–^* mice (38, 51). Therefore, caspase-8 and its enzymatical activity play a critical role in the regulation of apoptosis, necroptosis, and pyroptosis, a type of inflammasome-mediated cell death.

There is also a strong evidence that RIPK1 is an essential substrate of caspase-8 as mutant *Ripk1^D325A/D325A^* mice, in which the caspase-8 cleavage site Asp325 was abolished, died between E10.5 and E11.5. The embryonic lethality of *Ripk1^D325A/D325A^* mice can be prevented by inhibition of RIPK1 kinase activity, deletion of *Tnfr1*, or a combined deletion of *Fadd* and *Ripk3*, or deletion of *Fadd* and *Mlkl* (14, 38, 39). Although *Ripk1^D325A/+^* and kinase-dead *Ripk1^D138N,D325A/D138N,D325A^* mice survived, they developed multi-organ inflammation, and their cells were hypersensitive to TNF-induced cell death (14, 38). Therefore, cleavage of RIPK1 by caspase-8 is a mechanism for preventing abnormal cell death.

These functional and mechanistic studies of RIPK1 in mouse have provided compelling evidences for the indispensable role of RIPK1 in development and inflammation. Besides, they have established platforms for the investigation of RIPK1-associated diseases in humans.

## RIPK1-Related Human Immune Diseases

Dysregulation in the innate immune system can result in immunodeficiency or autoinflammatory disease. Immunodeficiency is a state in which the immune system’s ability to defend against various pathogens is diminished or severely compromised. Systemic autoinflammatory diseases result from the over-activation of innate immune cells, such as circulating and tissue-resident myeloid cells. Given that these are life-long immunological disorders, dysregulation in the adaptive immune system can ensue. From 2018 till now, six independent groups have reported patients with RIPK1-associated immunodeficiency or autoinflammatory diseases (9–14). Patients with RIPK1 deficiency and RIPK1 non-cleavable mutations present with distinct clinical manifestations. The summary of the genotypes and clinical phenotypes of 14 RIPK1-deficiency and 12 CRIA patients are listed in **Table 1**, and the position of pathogenic variants in RIPK1 are indicated in **Figure 1E**.

**Table 1:** Genotypes, clinical phenotypes, immunological manifestations and treatments of RIPK1-deficiency and CRIA patients.

Patient		Ethnic	Gender	cDNA change	Protein change	Age of onset	Manifestations										Treatments	Outcome
							CD3+/CD4+/CD8+ T/B	IBD/colitis	Hepato megaly	Spleno megaly	Arthritis/arthralgia	Pneumonia	Fevers	Lympha denopathy	Oral lesion/ulcers	Others		
**RIPK1-deficiency (recessive inheritance)**																		
family 1	**P1**	Pakistan	M	867_870delTTTA	Y289*	1 mo.	LLLR	Y	N	N	Y	Y	N	N	N	bronchiectasis, HCMV, recurrent RI	HSCT	Died
family 1	**P2**	Pakistan	M	867_870delTTTA	Y289*	1 mo.	RLRR	Y	N	N	Y	N	N	N	N	severe RSV bronchiolitis, RI	HSCT	Alive
family 2	**P3**	Arab	F	688_688+20del	N230Hfs*14	1 mo.	LLLL	Y	N	N	Y	Y	N	N	N	recurrent HSV1 infection	HSCT	Died
family 3	**P4**	Arab	F	460-133_689-244del	DELexon5	2 yrs.	LLLL	Y	N	N	Y	N	N	N	N	severe RSV bronchiolitis, recurrent OM	IVIG	Alive
family 4	**P5**	Caucasian	M	1844T>C	I615T	6 mos.	LLLR	Y	Y	Y	N	Y	N	N	Y	sepsis, esophagitis, gastritis, perianal disease	Ab, AZA, CS, IFX	Died
family 5	**P6**	Arab	F	1934C>T	T645M	1 mo.	RRRH	n.r.	n.r.	n.r.	N	Y	N	N	N	OM, U/LRI, perianal disease	Ab	n.r.
family 6	**P7**	Caucasian	F	1278C>A	Y426*	1 day	LLLL	Y	Y	Y	N	N	N	N	N	GI infections, U/LRI, septicemia, hepatitis	CS, HSCT	Died
family 7	**P8**	Arab	F	954delG	M318Ifs*194	1 day	LLLL	Y	N	N	N	N	N	N	N	esophagitis, gastritis, UTI, perianal disease	n.r.	Died
family 8	**P9**	Arab	F	1934C>T	T645M	1 day	n.r.	Y	N	N	Y	Y	N	N	Y	OM, esophagitis, UTI, perianal disease	Ab, AZA, CS, IFX	Alive
family 9	**P10**	North Africa	M	1802G>A	C601Y	6 mos.	LLRL	Y	N	N	N	Y	N	N	Y	OM, perianal disease, omphalitis	Ab, AF	Alive
family 9	**P11**	North Africa	M	1802G>A	C601Y	20 days	LLLL	Y	N	N	N	Y	N	N	Y	OM, tetany, perianal disease	Ab, AF	Alive
family 9	**P12**	North Africa	F	1802G>A	C601Y	3 mos.	LLLL	Y	N	N	N	N	N	N	Y	OM, tetany, perianal disease	Ab, AF, CS	Alive
family 10	**P13**	South America	M	636C>G	Y212*	1 day	LLLL	Y	Y	Y	N	N	N	N	N	esophagitis, gastritis	n.r.	Alive
family 11	**P14**	Chinese	M	998C>A 1934C>T	S333* T645M	3 mos.	HLHL	Y	Y	Y	N	Y	Y	n.r.	Y	anemia, jaundice, perianal abscess, anal fistula, malnutrition	IVIG, Ab	Died
**CRIA syndrome (dominant inheritance)**																		
family 1	**P1**	n.r.	F	970G>A	D324N	2 mos.	n.r.	N	N	Y	N	N	Y	Y	N	abdominal pain, tonsilitis	Anti-IL-6R	Alive
family 2	**P2**	Italian	F	970G>C	D324H	Birth	RLHL	N	N	N	N	N	Y	Y	N		Anti-IL-6R	Alive
family 2	**P3**	Italian	M	970G>C	D324H	2 wks.	RRRR	N	N	N	Y	N	Y	Y	Y	abdominal pain	Anti-IL-6R	Alive
family 2	**P4**	Italian	F	970G>C	D324H	Birth	RRRR	N	N	N	N	N	Y	Y	Y		Anti-IL-6R	Alive
family 2	**P5**	Italian	M	970G>C	D324H	Birth	HLHR	N	Y	Y	N	N	Y	Y	Y		n.d.	Alive
family 2	**P6**	Italian	F	970G>C	D324H	Birth	RLRR	N	Y	Y	Y	N	Y	Y	Y	abdominal pain, tonsilitis	Anti-IL-6R	Alive
family 3	**P7**	n.r.	M	970G>T	D324Y	6 mos.	RRRR	N	N	Y	Y	N	Y	Y	Y	abdominal pain, tonsilitis	Anti-IL-6R	Alive
family 4	**P8**	Chinese	M	971A>T	D324V	2 mos.	RRRH	N	N	N	N	N	Y	Y	N	abdominal pain, microcytic anemia	Anti-IL-6R	Alive
family 5	**P9**	Canadian	F	970G>C	D324H	6 mos.	n.r.	N	Y	Y	N	N	Y	Y	N	microcytic anemia	n.d.	Alive
family 5	**P10**	Canadian	M	970G>C	D324H	1 mo	n.r.	N	N	Y	N	N	Y	Y	N	microcytic anemia	n.d.	Alive
family 5	**P11**	Canadian	M	970G>C	D324H	n.r.	n.r.	N	N	N	N	N	N	N	N	microcytic anemia	n.d.	Alive
family 5	**P12**	Canadian	M	970G>C	D324H	1 mo	n.r.	N	N	Y	N	N	Y	Y	N	microcytic anemia	n.d.	Alive

AA, amino acid; Ab, antibiotics; AF, antifungal; AZA, azathioprine; CS, corticosteroids; IBD, inflammatory bowel disease; IFX, infliximab; IVIG, intravenous immunoglobulin; HCMV, human cytomegalovirus; HSV1, herpes simplex virus 1; n.r., not reported; n.d., not done; OM, otitis media; RSV, respiratory syncytial virus; TPN, total parenteral nutrition; U/LRI, upper and lower respiratory tract infections; UTI, urinary tract infection; Y, yes; N, no; Values above, below and within the reference ranges are marked as ‘H’ (high), ‘L’ (low) and ‘R’ (regular), respectively.

### Immunodeficiency and Inflammation Caused by RIPK1 Deficiency

Unlike postnatal lethality of *Ripk1* constitutive knockout mice, patients with *RIPK1* biallelic LoF mutations were born alive, however, they suffered from severe and potentially lethal immunodeficiency (9–12), and one of them had autoinflammatory manifestations (12). The associated phenotypes include recurrent viral, bacterial, and/or fungal infections, early-onset inflammatory bowel disease (IBD), and progressive polyarthritis. The immunodeficiency results from impaired differentiation of T and B cells, profound lymphopenia, and decreased production of proinflammatory cytokines including IL-6, TNF and IL-12. The inflammatory component of this disease is possibly related to the activation of NLRP3 inflammasome and high production of IL-1β cytokine (**Figure 1C**). This observation requires further investigations.

### Autoinflammation Caused by Non-Cleavable RIPK1 Variants

Patients with heterozygous missense mutations at the RIPK1 residue Asp324 present with recurrent fevers and lymphadenopathy (13, 14). The caspase-8 cleavage site Asp324 is highly conserved across species, indicating its importance from the evolutionary prospect. The disease-associated variants Asp324Val, Asp324His, Asp324Asn, and Asp324Tyr were identified in different families either as a *de novo* mutation or as a dominantly inherited allele. These pathogenic variants render RIPK1 non-cleavable, as they block caspase-8 mediated cleavage, thereby promoting RIPK1 activation.

## Molecular Mechanisms and Therapeutic Attempts

### Impaired Inflammation Pathways and Disordered Cytokine Production

Interestingly, both RIPK1-deficiency and CRIA patients present with a substantial dysregulation in inflammatory pathways and cytokine production. These cellular phenotypes were replicated in RIPK1-deficient cell lines (9, 10) and RIPK1 Asp325 mutation knock-in mouse embryonic fibroblasts (MEFs) (13, 14).

Biallelic LoF mutations in RIPK1 result in reduced NF-κB and MAPK signaling. Fibroblasts from a RIPK1-deficiency patient (c.688_688+20del, p.Asn230Hisfs*14) had markedly reduced phosphorylation of p38 and AP-1 subunit c-Jun, as well as partially reduced phosphorylation of NF-κB p65 under TNF or poly(I:C) stimulation, which showed impaired proinflammatory signaling downstream of TNFR1 or TLR3 (10). Similarly, *RIPK1*-deficient cell lines (HCT-116, Jurkat) showed reduced NF-κB activation in response to TNF (9). Consequently, TNF-induced secretion of IL-6 and RANTES in patients’ fibroblasts, LPS-induced production of IL-6, TNF, and IL-12 in patient’s primary monocytes, and LPS-induced IL-6 and IL-10 in *RIPK1* ^-/-^ THP1 cells were all significantly diminished (9).

RIPK1-deficient patients have dysregulated T cell responses under PHA stimulation with reduced production of IL-17 and IFN-γ, while the levels of TNF, IL-6, and IL-10 were normal. In contrast, IL-1β production was markedly increased in PHA-stimulated patient’s blood and LPS-stimulated *RIPK1* ^-/-^ monocytic cell lines (9, 10). These observations were in agreement with previous studies reporting an altered inflammasome activity in conditional *Ripk1* knockout mice (52). The altered IL-1β release has been associated with increased NLPR3 activity and MLKL-dependent necroptosis, based on evidence that inhibitors of NLRP3 or MLKL can reduce IL-1β secretion in LPS-stimulated RIPK1-deficient cell lines (BLaER1 and THP1 cells) (9, 10).

In contrast, CRIA patients’ PBMCs and monocytes have a strong inflammatory signature in NF-κB and type I IFN pathways, and elevated production of IL-6, TNF, and IL-1β cytokines (13). Similarly, mouse models and cell lines engineered with the CRIA-associated mutations display enhanced inflammatory response induced by TNF (13, 14). However, there exist cell-specific effect of non-cleavable RIPK1, as loss of RIPK1 cleavage in patient-derived fibroblasts did not affect TNF-induced NF-κB activation, and *Ripk1* knock out MEFs complemented with non-cleavable *Ripk1* variants had no difference in NF-κB signaling activation upon TRAIL stimulation (13, 14).

Another difference between the CRIA and RIPK1-deficient patients is the inducer of fluctuated cytokines. The reduced secretion of IL-6 in *RIPK1* ^-/-^ THP1 cells is independent of necroptosis since it can’t be restored by adding necroptosis inhibitor (10). Whereas, the cytokine increases observed in the CRIA patients’ PBMCs or in *Ripk1* ^-/-^ MEFs complemented with RIPK1 Asp325 mutants were dependent on RIPK3 and caspase-8 (13, 14), suggesting that cell death is the major contributor to cytokine induction in the context of non-cleavable RIPK1.

### Dysregulation of Cell Death

RIPK1 regulates both apoptosis and necroptosis by forming different complexes. However, controversy exists on the specific mechanism based on different observations in various cell lines.

Cuchet-Lourenço et al. found that RIPK1-deficient patient-derived fibroblasts were more sensitive to TNF or poly(I:C) induced cell death, which was reversed by complementation with wild-type RIPK1 (10). Further experiments revealed increased phosphorylation of RIPK3 and MLKL with no cleavage of caspase-8 in patients’ fibroblasts in response to poly(I:C) stimulation. In addition, the MLKL inhibitor NSA and RIPK3 inhibitor GSK’872 can rescue patients’ cells from poly(I:C)-induced cell death, whereas the pan-caspase inhibitor zVAD-fmk and RIPK1 inhibitor Nec-1s had no effect. Together, these findings indicate that necroptosis was a major cell death mechanism in RIPK1-deficiency disease. A similar cell death phenotype was observed in *RIPK1^−/−^* THP1 cells.

In parallel investigations, Yue Li et al. used *RIPK1^-/-^* HT-29 cells to investigate the effect of RIPK1 deficiency on TNF-induced cell death (9). In contrast to patient-derived fibroblasts and myeloid cell lines that were prone to necroptosis induced by poly(I:C), *RIPK1^-/-^* HT-29 cells showed resistance to TNF and BV6 induced apoptosis and to TNF, BV6 plus zVAD-fmk induced necroptosis (**Figure 1C**).

In CRIA patients, the non-cleavable RIPK1 initiates RDA and necroptosis in myeloid cells but not in patient-derived fibroblasts (13, 14). Panfeng Tao et al. reported that patients’ PBMCs displayed increased sensitivity to apoptosis (induced by co-treatment with TNF and apoptosis-inducing SMAC mimetic SM-164, T/S) and necroptosis (induced by co-treatment with SM-164 and zVAD-fmk, S/Z). This phenotype was suppressed by RIPK1 inhibitor Nec-1s, indicating that RIPK1 kinase activity may promote downstream cell death pathways. Evidence of necrotic cell death *in vivo* was demonstrated by detecting cyclophilin A in a patient’s urine samples during the disease flare. Similarly, TNF-induced cell death was elevated in *Ripk1^-/-^* MEFs expressing mutant RIPK1 and was inhibited by Nec-1s or the RIPK1 kinase inactivating mutation Asp138Asn. The activated RIPK1 can drive RDA independent of necroptosis as *Ripk1^D325A/D325A^Ripk3^−/−^* MEFs remained sensitized to T/S induced apoptosis (13, 14).

The work by Lalaoui et al. provides a comprehensive *in vitro* and *in vivo* description of the molecular mechanisms of inflammation in CRIA (14). Besides the findings that MEFs with RIPK1 non-cleavable mutants are sensitized to TNF-induced apoptosis and necroptosis, this study reports that caspase-8 mediated RIPK1 cleavage during embryogenesis can inhibit necroptosis and maintain normal development. As to the role of RIPK3, this study shows that the inhibition of RIPK3 kinase activity had little effect on the induction of cell death, while genetic loss of *Ripk3* in *Ripk1^D325A/D325A^* MEFs had significantly reduced auto-phosphorylation of RIPK1 and caspase activation under T/S treatment. These data suggest that RIPK3 contributes mainly in a structural capacity to the activation of caspase-8.

Taken together, studies on the mechanisms of CRIA syndrome revealed that non-cleavable variants in RIPK1 promote cell death as well as the production of pro-inflammatory cytokines.

### Clinical Treatments

Better understanding of upstream and downstream events in the RIPK1 activation and signal transduction process will help to implement targeted treatment for patients with *RIPK1* pathogenic mutations. For patients with CRIA, the small molecule RIPK1 inhibitor might be a treatment choice (53, 54). Although cytokine inhibition is effective in many autoinflammatory diseases, such as anti-IL-1β therapies for most inflammasomopathies (55, 56), TNF inhibitors for NF-κB-activation disorders (57, 58), and JAK inhibitors for interferonopathies (59), concerns remain on the long-term use and efficacy of biological therapies. CRIA patients appear to be responsive to therapy with an IL-6R antagonist (13, 14). In patients with RIPK1 deficiency, treatment with IL-1 inhibitors may ameliorate the inflammatory manifestations. Hematopoietic stem cell transplantation (HSCT) remains another possibility as it can be curative in patients with severe immunodeficiencies, if performed early-in life (60–62). HSCT has resolved the inflammatory manifestations and reduced the frequency of infections in one RIPK1-deficient patient (10). Gene therapies, whether to replace deficient *RIPK1* gene or gene-editing therapy in CRIA patients, might be a long-term consideration.

The summary of treatments used thus far in RIPK1-deficiency and CRIA patients is shown in **Table 1**. The response and outcomes to different therapies have been highly variable among patients, and the long-term consequences remain to be described.

## The Role of RIPK1 in Other Autoinflammatory and Autoimmune Disorders

Pathogenic mutations in genes encoding multiple regulators of the NF-κB pathway and the RIPK1 activation, including TNFR1, NEMO, A20, LUBAC and OTULIN, have been found in patients with autoinflammatory and autoimmune diseases (63). The identification of these disorders has contributed to a better understanding of mechanisms that underlie the regulation of the canonical NF-kB pathway in humans.

TNFR1 is the major signaling receptor of TNF that can initiate a number of downstream pathways including NF-κB, MAPK signaling and programmed cell death. These processes are tightly connected with RIPK1 scaffolding function and partially dependent on RIPK1 kinase activity. Pathogenic missense mutations in the extracellular domain of TNFR1 (encoded by *TNFRSF1A)* causes TNF Receptor-associated Periodic Syndrome (TRAPS), an autosomal dominant autoinflammatory disease (64). The clinical manifestations of TRAPS include periodic long-lasting fever, serositis, abdominal pain, arthralgia, migratory skin rash, and myalgia (65). The molecular pathogenesis of TRAPS is still elusive, and the contribution of cell death pathways have not been investigated. The mutant TNFR1 proteins accumulate in the cells, which can trigger endoplasmic reticulum (ER) stress and unfolded protein response (UPR) (66), as well as excessive mitochondrial reactive oxygen species (mROS) (67). All these processes are known to promote inflammatory cytokine production and can explain a strong inflammatory signature in this disorder.

Mutations in NEMO, an essential modulator of NF-κB encoded by *IKBKG*, are associated with incontinentia pigmenti and X-linked ectodermal dysplasia with immunodeficiency (68). NEMO serves as an adaptor protein to recruit IKKα/β for NF-κB activation and TBK1/IKKϵ for type-I IFN signaling, all of them are negative regulators of RIPK1 kinase activation and its kinase dependent cell death as well (27). Individuals with hypomorphic or LoF mutations in *IKBKG* gene develop severe immunodeficiency, ectodermal dysplasia manifesting with coning teeth, hypodontia and inability to sweat, and colitis (69). Ectodermal dysplasia can be explained by the inability of the ectodysplasin A receptor (a TNF receptor family member) to induce NF-kB activation  (69).

A20, encoded by *TNFAIP3*, is an enzyme that regulates NF-κB activation by cleaving K63-linked polyubiquitin chains on RIPK1 and other components in the complex I. It can also build K48-linked polyubiquitin chains on RIPK1 (28). A20 contains an N-terminal ovarian tumor (OUT) domain which deubiquitylate K63-polyubiquitin chains, and seven C-terminal zinc finger (ZnF) domains that bind to ubiquitin and act as an E3-ligase, of which ZnF7 can bind and modulate linear ubiquitin chains (70). Heterozygous LoF mutations in *TNFAIP3* gene are associated with Haploinsufficiency of A20 (HA20), a childhood-onset systemic autoinflammatory disease manifesting with recurrent fever, arthritis, oral and/or genital ulcers, skin allergies, gastrointestinal inflammation, eye inflammation and autoimmunity (71, 72). HA20 patients’ PBMCs and fibroblasts have accumulated K63-linked polyubiquitin chains, as well as activation of NF-κB and MAPK pathways with increased production of many pro-inflammatory cytokines, such as TNF, IL-1β and IL-6.

The LUBAC complex is involved in the activation of NF-κB pathway in response to TNFR1 signaling by generating linear ubiquitin chains on NEMO, RIPK1, and other proteins. Biallelic LoF mutations in two of the LUBAC component: HOIL-1 (heme-oxidized IRP2 ubiquitin ligase 1; *RBCK1*) or HOIP (HOIL-1 interacting protein; *RNF31*), cause LUBAC deficiency, an autosomal recessive disease characterized by severe immunodeficiency, recurrent fever, and polyglucosan myopathy (73, 74).

OTULIN-related autoinflammatory syndrome (ORAS), also known as otulipenia, is an autoinflammatory disease caused by biallelic LoF mutations in the linear deubiquitinase OTULIN (75, 76). Patients present with early-onset recurrent fever, skin rash, neutrophilic nodular panniculitis/lipodystrophy, and gastrointestinal inflammation (76). The pathogenic variants reside in the OTU domain, which has a deubiquitination enzymic activity, and affect the binding of OTULIN to the linear ubiquitin chains. The excessive accumulation of poly-linear ubiquitin chains in patients’ PBMCs and fibroblasts causes constitutive activation of canonical NF-κB signaling pathway, and excessive production of pro-inflammatory cytokines (75, 76).

## Broadened Roles of RIPK1 in Regulating Multiple Pathways

Remarkable insights on the RIPK1 function in human immune diseases have been revealed during the past few years and are summarized in the following section.

First, RIPK1-deficient monocytes exhibit increased sensitivity to necroptosis and IL-1β release upon LPS stimulation. Previously, Gaidt, et al. have identified an alternative inflammasome signaling pathway *via* TLR4-TRIF-RIPK1-FADD-Caspase-8 in human monocytes (77). The LPS-dependent inflammasome signaling was only observed in the RIPK1-deficient monocytes transdifferentiated from immortalized human B cells but not in mouse models. Cuchet-Lourenço et al. identified elevated IL-1β production by T cells *in vivo* in the context of RIPK1 deficiency (10). In addition, previous studies have shown that RIPK1 and RIPK3 appears to promote the activation of the NLRP3 inflammasome independent of cell death, and this effect can be suppressed by caspase-8 (78).

Second, CRIA patient’s fibroblasts display resistance to both necroptosis and another kind of programmed cell death, ferroptosis (13), which is an iron-dependent process accompanied by excessive lipid peroxidation (79). This phenomenon is opposite to that observed in patient’s PBMCs and might explain why there is no skin involvement of CRIA patients. This finding not only provides new evidence for heterogeneity of cell functions with respect to the cell type, but also makes a direct link between non-cleavable RIPK1 variants to ferroptosis. RIPK1 kinase activation can also be promoted by reactive oxygen species (ROS) production, the mediator of ferroptosis (80). However, ferroptosis is biochemically distinct from other programmed cell death pathways and can be triggered by a unique set of inducers (81). The role of RIPK1 in the regulation of ferroptosis needs to be investigated further.

## Future Research

Despite enormous progress in understanding the role of RIPK1 in mediating cell death and inflammation, many questions remain concerning the molecular pathogenesis of RIPK1-associated diseases. They are following: 1) the unexpected difference in responses of CRIA patient’s fibroblasts and PBMCs to the same cell death inducers; 2) the causal relationship between RIPK1 non-cleavable variants and the expression of ferroptosis- or antioxidant-related genes; 3) the influence of RIPK1 deficiency on cell death *in vivo*; 4) the relationship between elevated expression of cytokines and increased cell death in CRIA patients. These questions might, in part, be answered with the identification of new pathogenic mutations in human patients that result either in hyperactivation or deficiency of RIPK1. Ultimately, answering these questions will help develop more efficient and targeted treatments for patients with RIPK1-associated diseases.

Of particular interest is the functional cell-type heterogeneity in PBMCs and fibroblasts of CRIA patients. Transcriptional analysis revealed decreased expression of RIPK1 and TNFR1, high levels of the antioxidant glutathione (GSH), and low levels of ROS in patients’ fibroblasts (13) (**Figure 1D**). These observations may in part explain the different cellular phenotypes between patient fibroblasts and PBMCs. Fibroblast is a type of resident permanent cells, and not like short-life bone-marrow-derived inflammatory cells such as granulocytes and macrophages, that can be eliminated quickly to downregulate inflammation (82). Previous studies have found that fibroblasts play a critical role in the switch of acute resolving inflammation to chronic persistent one by modifying the quantity and duration of the inflammatory infiltrate (83).

## Discussion

RIPK1, as a critical regulator of NF-κB signaling, has the ability to transmit and interpret extracellular stimuli such as TNF to activate different downstream signaling pathways including NF-κB activation, apoptosis and necroptosis, with distinct outcomes.

The recent discovery of RIPK1-deficiency (9–12) and CRIA (13, 14) have enhanced the understanding of the molecular mechanisms of RIPK1 function in humans. Although the pathological mechanisms of these diseases have been revealed partly, there still exist many unsolved questions. A better understanding of the inflammatory mechanisms of rare RIPK1-associated monogenic diseases may help to develop more targeted therapies that could be used for a series of diseases, including rare neurodegenerative, autoimmune (psoriasis, ulcerative colitis and arthritis), acute ischemic diseases and other conditions such as sepsis and pancreatic ductal adenocarcinoma (54, 84).

## Author Contributions

JZ and TJ contributed equally. JZ wrote the original draft and draw the figure. TJ provided valuable comments and modified the manuscript. IA and QZ reviewed and approved the final version of the manuscript. All authors contributed to the article and approved the submitted version.

## Conflict of Interest

The authors declare that the research was conducted in the absence of any commercial or financial relationships that could be construed as a potential conflict of interest.
